# Integratable quarter-wave plates enable one-way angular momentum conversion

**DOI:** 10.1038/srep24959

**Published:** 2016-04-22

**Authors:** Yao Liang, Fengchun Zhang, Jiahua Gu, Xu Guang Huang, Songhao Liu

**Affiliations:** 1 Guangdong Provincial Key Laboratory of Nanophotonic Functional Materials and Devices, South China Normal University, Guangzhou 510006, China; 2 Centre for Micro-Photonics, Faculty of Science, Engineering and Technology, Swinburne University of Technology, Hawthorn, Victoria 3122, Australia; 3 Institute of Opto-Electronic Materials and Technology, South China Normal University, Guangzhou 510631, China

## Abstract

Nanophotonic waveguides are the building blocks of integrated photonics. To date, while quarter-wave plates (QWPs) are widely used as common components for a wide range of applications in free space, there are almost no reports of Integratable QWPs being able to manipulate the angular momentum (AM) of photons inside nanophotonic waveguides. Here, we demonstrate two kinds of Integratable QWPs respectively based on the concept of abrupt phase change and birefringence effect. The orientation of the equivalent optical axis of an Integratable QWP is designable. Remarkably, a combination of two integratable QWPs with different equivalent optical axes leads to an integrated system that performances one-way AM conversion. Moreover, this system can be used as a point source that can excite different patterns on a metal surface via directional excitation of surface plasmon polaritons (SPP). These results allow for the control of AM of light in nanophotonic waveguides, which are crucial for various applications with limited physical space, such as on-chip bio-sensing and integrated quantum information processing.

Polarization and phase are fundamental properties of light. They often play an important role in manipulating the angular momentum (AM) of photons, since the spin part (SAM) of AM is associated with the circular polarization, while the orbital part (OAM) is related to the spatial phase distribution of photons^1^,^2^,^3^. The chiral light carrying AM is of widespread interest, and has been used to study a variety of applications, such as optical trapping^4^, biosensing^5^, quantum information science^6^,^7^ and laser cooling^8^.

Typically, such chiral lights are created by using quarter-wave plates (QWPs)^9^, spiral phase plates^10^, cylindrical lens mode converters^11^ and more generally spatial light modulators. However, those components are relatively large in size ranging from hundreds of micrometers to several centimeters and, therefore, they are not ideal candidates regarding to large-scale integration. A reduction in the size of optical components while maintaining a high level of performance is, so far, a key challenge for integrated photonics. Novel metallic and dielectric nanostructures, such as metasurfaces^12^,^13^,^14^ and nanophotonic waveguides based devices^15^,^16^, provide a powerful solution to this challenge. Interestingly, light fields confined in nanophotonic waveguides exhibit remarkably spin-orbit interaction such that their spin and orbital AM get coupled^17^,^18^,^19^. Consequently, a change in the polarization state of the guided mode in a nanophotonic waveguide will inevitably change the spatial phase distribution and even the mode’s intensity profile. In this way, steering the phase and polarization of light fields inside nanophotonic waveguides offers potential to achieve novel optical phenomena such as spin-controlled unidirectional emission of photons^16^ and extraordinary spin AM states that being neither parallel nor perpendicular to the direction of propagation (see Supplementary Information).

Here we present two kinds of quarter-wave plates (QWPs) with different optical axis (slow axis) orientations for integrated silicon photonics (ISPs), which are able to manipulate the AM of light inside nanophotonic waveguides. These devices are ultra-compact with only a few micrometers in length and compatible with conventional complementary metal-oxide-semiconductor (CMOS) technology. In particular, a combination of these QWPs leads to a metallic-silicon waveguide system that performs AM conversion in only one direction (Fig. 1a). This system can be used as a point source that can excite a two dimensional (2D) dipole or an in-plane spin-dipole via near-field excitation of surface plasmon polaritons (SPP) in thin metallic films. These results may bring new opportunities for integrated quantum information computing and near-field optical manipulation.

## Results

### Optical performance of one-way AM conversion

**Figure 1:**
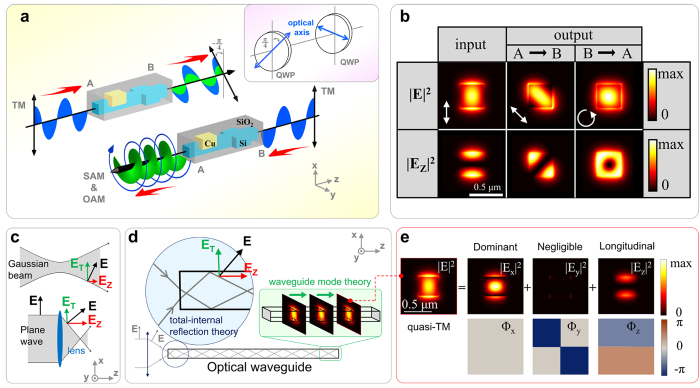
(**a**) Schematic of the proposed system: a quasi-TM mode converts into another quasi linearly polarized mode in the forward propagation (A to B), with the dominant electric field component rotated about −π/4; yet the same quasi-TM mode transforms into a quasi-circularly polarized mode with a longitudinal optical vortex component in the counter propagation (B to A). The analogy of the proposed system: a combination of two QWPs with different optical axis orientations (inserted figure). The coordinate system used. QWP, quarter-wave plate. (**b**) Optical intensities (|E|^2^) of the input and output modes, and their longitudinal polarized components. The arrows indicate the polarization states at the central point of the input and output Si square waveguides. (**c**) The sources of longitudinal light field component (E_z_) in free space: diffraction and focusing of light. Schematic of the relationship between the transverse component (E_T_) and E_z_. (**d**) The schematic drawing of light travelling in a nanophotonic waveguide, where strong E_z_ can be attained. Two inserted figures are drawn according to the total-internal reflection theory and waveguide mode theory, respectively. (**e**) The decomposition of a quasi-TM mode in Si waveguide of a rectangle core (340 nm * 340 nm). The intensity (|E|^2^) and phase profiles (Φ) of each component are shown accordingly. The operating wavelength is 1.55 μm.

Figure 1a shows the functionality of the proposed system: one-way AM conversion. When light travels from port A to port B, a fundamental quasi-TM mode, whose predominant polarization component points along the x-axis, transforms into another quasi-linearly polarized mode with the dominant polarization component orienting along (*φ* = −*π/4*)-direction, where *φ* is the angle down from the positive x-axis. Also, a longitudinal polarized component (**E**_**z**_) is generated owing to the strong mode confinement in the nanophotonic waveguide, as shown in Fig. 1b. On the other hand, when light travels in the opposite direction (B to A), the same quasi-TM mode transforms into a novel quasi-circularly polarized mode with a longitudinal optical vortex component. The underlying physical mechanism that enables the generation of a longitudinal optical vortex is spin-orbital conversion, which can occur when the light fields are strongly confined in the transversal direction^2^, i.e., being focused by a high numerical aperture (NA) lens^20^ or confined in a nanophotonic waveguide (our case). In this non-paraxial regime, the spin and orbital angular momentum of photons are inevitably coupled together instead of being independent physical quantities^17^,^18^. To some extent, our system is analogous to two QWPs with difference optical axis orientations in free space (inserted figure in Fig. 1a), regarding to the polarization state at the central point of the silicon (Si) square waveguide.

### Longitudinal electric field component (E_z_)

In our finite-different time-domain (FDTD) simulations, only the fundamental 0^th^ order modes are considered, since high order modes of interest are restrained in the Si square waveguide at *λ* = *1.55* *μm*. Due to the strongly transversal confinement, the light fields supported by such nano-waveguides are not purely transversal and, instead, a longitudinal polarized component that points to the direction of the propagation of light (z-axis) is generated. The longitudinal polarized component holds great promises for the investigation of many novel physical phenomena such as spin to orbital AM coupling^20^ and Möbius strips of optical polarization^21^, and has been applied to a variety of applications, ranging from optical storage^22^ to particle acceleration^23^.

A fundamental guided mode can be mainly decomposed into a dominant transversal component (**E**_**T**_) and a longitudinal component (**E**_**z**_), that is **E** = **E**_T_ + **E**_z_, and the relationship between them is given by^24^,^25^,


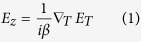


where *β* is the propagation constant and ∇_*T*_ the transverse gradient. Equation 1 indicates that the **E**_**z**_ results from the gradient of **E**_**T**_, and it implies that the polarization state of photons is position-dependent in the transverse plane (xy-plane) of the Si square waveguide. The underlying physics of this phenomena is spin-orbit interaction^17^,^18^,^19^. In particular, the light field at the central point of the Si square waveguide is purely transversal regarding to a fundamental 0^th^ order mode, where the amplitude of the dominant polarization component reaches its peak while the one of the longitudinal component equals zero (see Supplementary Information). Thus, it is reasonable to use the polarization state at the central point to represent the dominant polarization and phase of a certain fundamental mode, and this method will be used throughout this paper. Moreover, we mainly discuss the circularly 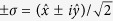 and *φ-*angle linearly 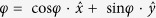 polarization, where the ± sign indicates the left (+) or right (−) handedness of circular polarization, *φ* the angle down from the positive x-axis, and 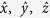 the unit vectors along the corresponding axes.

Ideally, only the polarization of infinite plane wave is purely transverse and, thereby, a longitudinal component (**E**_**z**_) can be found in light fields where the beam width is limited. Indeed, the longitudinal optical component, which originates from the spatial derivative of the transverse fields (**E**_**x**_, **E**_**y**_), is a nature of light^24^. The reason being is that light beams are usually diffractive, meaning that the beam does diffract and spread out as it propagates along the z-axis. Consequently, the longitudinal component occurs. An example to illustrate this point is a Gaussian beam in free space, the schematic of which is shown in Fig. 1c. But the **E**_**z**_ component is so small that it can be negligible in the paraxial case. An effective method to obtain a relative large **E**_**z**_ component is to get light beam strongly confined in the transverse direction, being focused via a high numerical aperture (NA) lens (Fig. 1c) or confined in a nanophotonic waveguide (Fig. 1d). Unlike the tightly focused beams in free space, where light diverges rapidly after the focal region, the light remains a constant mode distribution in any propagation distance when it travels in the nanophotonic waveguide, assuming there is no optical loss involved (Fig. 1d). For example, a quasi-TM mode, as shown in Fig. 1e, which can be mainly decomposed into a dominant transverse component (**E**_**x**_) and a longitudinal component (**E**_**z**_). Of course, there is a small portion of another transverse component (**E**_**y**_), which is so insignificant that it can almost be neglected (see Supplementary Information).

### Geometric details of the proposed system

**Figure 2:**
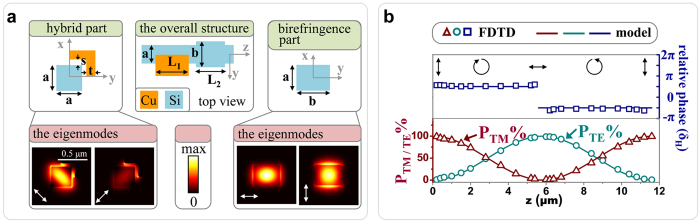
(**a**) Geometric details of the proposed system, and the associated eigenmodes supported in different waveguide sections, with the white arrows indicating the polarization states at the central point of Si core. (**b**) FDTD simulated (symbols) and analytical model (lines) dependences of polarized mode purity and relative phase of quasi TE- and TM-modes on the propagation distance in the hybrid waveguide section. The arrows indicate the polarization states at the central point of the connecting Si square waveguide.

There are two kinds of nanophotonic waveguides involved in our scheme, which corresponding to two integratable QWPs with different equivalent optical axis orientations (inserted figure in Fig. 1a). Besides, Si square waveguides are used at the input and output ports, and to connect these two QWPs. Figure 2a shows the geometric parameters of the propose system. The first part is a hybrid waveguide composed of an L-shaped copper (Cu) strip on the right top of the square Si core. The width and height of the Si core are equal, *a* = *340* *nm*. The spacer between the Cu strip and the Si core is *s* = *60* *nm*. The thickness of Cu strip is *t* = *220* *nm* and the overall length *L*_*1*_ = *2.8* *μm*. The second part is a birefringence Si waveguide, whose height and width are *a* = *340* *nm, b* = *440* *nm*, respectively, and the length *L*_*2*_ = *2.4* *μm*. The whole structure is surrounded by silica (SiO_2_), and the operating wavelength is 1.55 μm.

### Modes coupling in the hybrid waveguide section

We now consider the first hybrid waveguide section (Fig. 2a). Although the spatial mode distribution and the polarization of light are independent physical quantities in free space or in relative large waveguide structures^26^, they get connected in light fields that are strongly confined because of the spin-orbit interaction of light. Consequently, the guided mode’s polarization does indeed influence its spatial mode distribution in nanophotonic waveguides. To illustrate this point, we plot the fundamental modes supported in the hybrid waveguide in Fig. 2a, 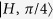 and 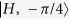, where *H* represents the hybrid waveguide and ±*π*/*4* are the corresponding polarized angles of the dominant polarization components (*φ* = ±*π/4*). The dominant polarization states of these two mode are 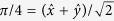 and 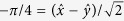, respectively.

The input quasi-TM mode 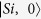 will firstly couple into another TM-like polarized mode 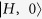 when light travels into the hybrid section. The mode 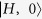 is obviously not the eigenmode that can remain stable during the propagation. Thus, the polarization state of the newly input mode 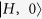 will gradually rotate, coupling into another orthogonally polarized mode, quasi-TE mode 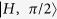, whose dominant polarization component points along the y-direction, and vice versa. By using the coupled mode approach, the light field dynamics in the hybrid waveguide are described by


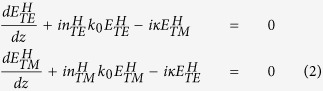


where 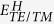 represent respectively field amplitudes of the two orthogonally polarized modes of 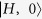 and 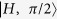 in the hybrid waveguide, 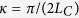 the coupling constant with coupling length *L*_*C*_, *k*_*0*_ = *2π/λ* the free space wavenumber, and 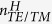 the effective indices for the two modes. Equations (2) can be solved analytically,


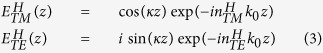


provided that 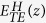 and 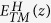 have the same normalization and 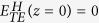, 
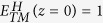
, where *z* is the propagation distance. Notably, there is an intrinsic *i* item for 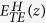, meaning that there is a relative phase lag of (*δ*_*H*_ = *π/2*) for the quasi-TM mode (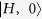) compared with the quasi-TE mode (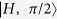), and this intrinsically abrupt phase lag remains a constant during the TE-TM coupling process in the first coupling period (*0* < *z* < *L*_*C*_). Surprisingly and in contrast to traditional bulk optics, where the phase change (*k*_*0*_*z*) relies on light propagation over distance^9^, the abrupt phase shift (*π/2*) is introduced as a result of the resonant (symmetric) nature of the L-shaped hybrid nanostructure. It should be emphasized that the initial conditions of the coupling equations have to be reset everytime after one quasi-linearly polarized mode is coupled into another. For example, the initial conditions will be changed as 
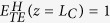
, 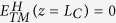 when the quasi-TM mode is converted into the quasi-TE mode, assuming there is no optical losses involved. In that case, there is a relative phase lag of π/2 for the quasi-TE mode compared with the quasi-TM mode in the next coupling period (*L*_*C*_ < *z* < *2L*_*C*_).

In Fig. 2b, we plot the theoretical and simulated results of polarized mode purity


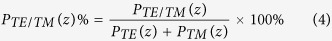


in the hybrid waveguide during the propagation of light, where 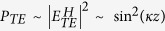 and 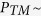 
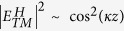
 are the modes’ energies. In a linearly system like the hybrid waveguide, the supported guided modes can be decomposed into the quasi-linearly polarized eigenmodes, 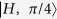 and 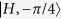, whose effective indices are respectively *2.445* + *0.00426i* and *2.315* + *0.0027i*. Thus, the light fields in the waveguide can be represented in the base of these modes, i.e., 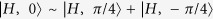 and 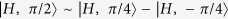. The coupling length *L*_*c*_, over which a 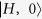 mode completely transforms into a 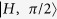 mode, can be expressed as:


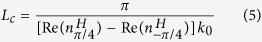


where 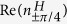 are the real parts of effective indices for the 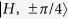 eigenmodes, and *k*_*0*_ the free space wavenumber. For the realization of a perfect integratable QWP that can generate a quasi-circularly polarized mode, both the amplitudes (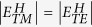) and relative phase (*δ*_*H*_ = *π/2*) conditions of the two orthogonal polarized modes should be simultaneously fulfilled. While the phase condition is already fulfilled due to the resonant nature of the L-shaped hybrid structure, the amplitude condition can be satisfied by engineering the length of the hybrid waveguide. It is desired to have equal amplitudes, 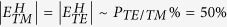, and thus, according to Equations (2, the required length is calculated to be *z* = (*L*_*c*_*/2*) ≈ *2*.98 μm, consistent with the FDTD simulation result of 2.80 μm (Fig. 2b).

**Figure 3:**
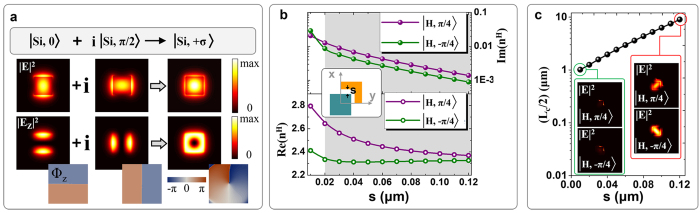
(**a**) The left handed quasi-circularly polarized mode consisting of a quasi-TM mode and a quasi-TE mode with a relative phase (π/2) between them. The electric field intensity (|E|^2^) and corresponding z-components and phases (|E_z_|^2^ and Φ_z_) of corresponding modes are shown. (**b**) The dependences of real and imaginary parts of effective indices on the the spacer (s) in the hybrid waveguide section for the two orthogonal modes (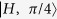 and 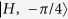). (**c**) The required length of the hybrid waveguide section (L_c_/2), for which a quasi-TM mode converts into a quasi-circularly polarized mode, versus the spacer (s). Inserted figures are the two orthogonal modes (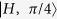 and 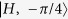) for different geometric parameter (s = 0.01, 0.12 μm). The operating wavelength is 1.55 μm.

After passing through the hybrid waveguide, the hybrid modes, 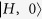 and 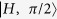, couple into Si square waveguide modes of 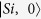 and 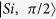
respectively, but the relative phase of *π/2* is preserved in the coupling process. Thus, the overall effect is that the hybrid waveguide converts an input quasi-TM mode 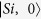 into a left-handed quasi-circularly polarized mode 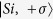, 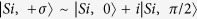 (Fig. 3a). The relative phase of *π/2* between 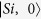 and 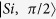 modes suggests that the quasi-circularly polarized mode does spin inside the Si square waveguide, spinning clockwise (from the point of view of the receiver). Importantly, the z-polarized vortex component of the 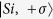 mode results from the spin to orbital conversion, and thus its twisted direction is coincident with that of the spin, clockwise (see Supplementary Information). Obviously, the first hybrid waveguide is functioning as an integratable QWP, whose equivalent optical axis oriented *π/4* (inserted figure in Fig. 1a). Interestingly, by engineering the length of the hybrid waveguide (*L*_*1*_), it is able to control the energy ratio between the two orthogonal polarized modes, 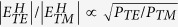, meaning that the equivalent optical axis of the integratable QWP is designable. In particular, when the length of hybrid waveguide is set to be *L*_*1*_ = *L*_*c*_, where the polarized mode purity 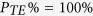, the hybrid waveguide is functioning as a TM-TE polarization rotator.

The spacer (*s*) is a key ingredient in our device. To evaluate the influence of the spacer on the performance of the hybrid waveguide section, in Fig. 3b, we plot the dependence of the effective index (real and imaginary parts) on the spacer for both 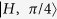 and 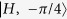 modes. For *s* > *0.02* *μm*, the imaginary parts [*Im(n*^*H*^)], which are associated with the propagation loss, decrease exponentially as the spacer increases, for both the orthogonal modes. However, the real parts [*Re(n*^*H*^)] seem to be a little disordered compared to the imaginary ones. Indeed, the real parts do indicate another exponential law for the proposed hybrid structure.

According to the supermode theory, the required length (*L*_*c*_*/2*) for the hybrid section, over which a quasi-TM mode (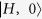) into a left-handed quasi-circularly polarized mode (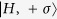), is given by,


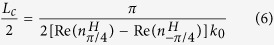


where 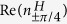 are the real parts of effective indices for the 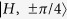 eigenmodes, and *k*_*0*_ the free space wavenumber. Figure 3c shows the dependence of *L*_*c*_*/2* on the spacer. An increase in the spacer leads to an exponential increase in the required length, which is solely determined by the real parts of the two orthogonal modes. Therefore, it is clear that there is a tradeoff between the propagation loss and the compactness of the hybrid waveguide section. We choose the spacer to be s = 60 nm in this work.

### The birefringence waveguide

The second waveguide involved in our scheme is a purely dielectric waveguide that exhibits large birefringence effect. In the Si square waveguide, the effective indices of both the quasi-TM 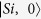 and -TE 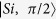 modes are equal, 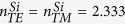 because of the diagonal symmetry of geometry. However, in the birefringence waveguide, the supported quasi-TM mode 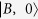 travels much faster than the quasi-TE mode 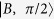, where *B* represents the birefringence waveguide and *0* and π/2 are the predominant polarization angles. Their effective indices are 2.476 and 2.642, respectively. Therefore, after passing through the birefringence waveguide, there is a relative phase *δ*_*B*_ between the two orthogonally polarized modes,


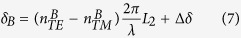


where 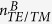 are the effective indices of two orthogonal modes, 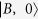 and 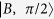, in the birefringence section, *L*_*2*_ the length of the waveguide, and Δ*δ* is a phase difference that caused by other factors, i.e., modes coupling between the Si square waveguide and the birefringence waveguide. According to Equation 7, the demand of a phase delay of π/2 requires *L*_*2*_ = *2.33 *μm, which coincides with the simulation result, *2.40* *μm*, assuming that 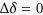. In this case, the birefringence waveguide works as an integratable QWP with an equivalent optical axis that points along the y-direction (inserted figure in Fig. 1a).

### The underlying physics of the one-way AM conversion

It is now clear that how this dielectric-metallic system functions: when light travels from A to B (Fig. 1a), the fundamental quasi-TM mode 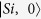 is firstly converted into a left-handed quasi-circularly polarized mode 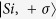 (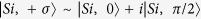) by the hybrid section. Next, the relative phase of π/2 between the 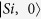 and 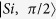
modes further enlarges to *π* by the birefringence waveguide, resulting in a *−π/4*-polarized quasi-linearly mode 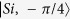 (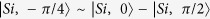) at the output port. In the counter propagation (B to A), however, the input quasi-TM mode 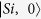 remains unchanged after passing through the birefringence waveguide, and next it is converted into a right-handed quasi-circularly polarized mode 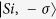 by the hybrid waveguide. Of course, similar things happen in the case of an input quasi-TE mode 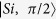, which will convert into a quasi-linearly polarized mode 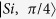 in the forward direction but a left-handed quasi-circularly polarized mode 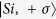 in the opposite direction. In quantum photonic circuits, information can be stored and processed via the AM states of photons and thus, this one-way AM behavior may open new opportunities for integrated quantum information processing.

The optical losses mainly arise from the modes coupling between different waveguide sections and the absorption loss of the hybrid waveguide section. The overall conversion efficiency of this QWPs system is around 86%, under the geometric parameters of *a = 340 nm, b = 440 nm, s  = 60 nm, t  = 220 nm, L 1 = 2.8 μm, L 2 = 2.4 μm* (Fig. 2a)

### The 2D dipole and the in-plane spin-dipole

**Figure 4:**
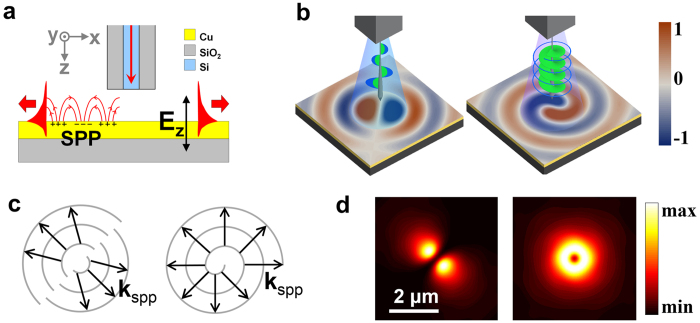
(**a**)Illustration of directional SPP excitation in near-field, where a nano Si square waveguide placed 400 nm away from a thin Cu film (50 nm thickness) with a SiO_2_ substrate. (**b**) The calculated normal component (*E*_*z*_) of SPP electric fields launched by a quasi linearly polarized mode 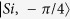 and a right-handed quasi-circularly polarized mode 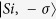, which are acting like a two dimensional dipole and an in-plane spin-dipole, respectively. (**c**) The generated SPP waves propagate radially away from the central point, with the wavefronts shown in gray. The SPP wave vectors (*k*_*SPP*_) are shown in black arrows. (**d**) Corresponding intensity (|E|^2^) distributions of the two modes on the surface of the Cu film. The near-field intensity patterns are dependent on the angular momentum states (phase and polarization related) of output modes.

The proposed system can be used as a point source that can excite different patterns on a metal surface, depending on the AM states of output modes. We place a 50 nm thickness Cu film (on silica substrate) 400 nm away from the output port (Fig. 4a). For directional SPP excitation on the surface of thin metallic films, usually only the component of the incident light that is perpendicular to the thin metal surface can be coupled into SPP^27^. The **E**_**z**_ component of the output mode is polarized perpendicular to the Cu film. Fortunately, the **E**_**z**_ is very strong in the output Si square waveguide, whose overall amplitude is as high as around 78% of that of the transversal component **E**_**T**_ (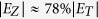). Interestingly, light fields in the Si square waveguide are tightly confined to a spot with a diameter much smaller than the corresponding wavelength of SPP. Its Fourier transform hence comprises a wide range of wave vector components, including those that match up with the wave vector of SPP (k_SPP_)^28^.

In the case of a quasi-linearly polarized output mode, i.e., 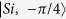, the SPP emission pattern on the Cu surface is approximately that of a 2D dipole (Fig. 4b). However, in the case of a quasi-circularly polarized mode, i.e., 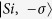, the SPP emission pattern is a novel in-plane spin-dipole that spinning while propagating radially away from the central point. Therefore, the proposed system can potentially be used as an SPP point source that can excite different patterns on a metal surface, depending on the AM states of the output modes. Figure 4c shows the wavefronts and SPP wave vectors of the two patterns. The intensity distribution profiles of the two SPP radiations are shown in Fig. 4d. It should be emphasized, however, that there are still a proportion of light fields cannot coupled into SPP, since their wave vectors mismatch with the *k*_*SPP*_, which accounts for the non-pure-zero intensity distribution at the central point of the SPP radiation profiles. The simulation characterization results of the near-field directional SPP excitation are represented in Supplementary Movie S1.

## Discussion

In conclusion, we demonstrated two kinds of ultra-compact QWPs that address the integrated challenge by offering a substantial reduction in size while allowing high level control over the AM of light inside nanophotonic waveguides. This is achieved by handling the polarization and relative phase between two orthogonally polarized modes. Importantly, a combination of these two QWPs results in a direction-dependent system that performs one-way AM conversion. The underlying physics holds fundamental potential and applicability to a wide range of applications, such as direct excitation of SPPs, detection of chiral molecules and nanoscale optical manipulation. It also opens exciting perspectives for integrated computing science and on-chip processing of quantum information, including AM-entangled optical qubits.

## Methods

The numerical experiments in this work were carried out by using the finite-different time-domain (FDTD) method (FDTD Solutions package from Lumerical Inc.). We apply perfectly matching layer (PML) boundary conditions in all directions. The 3D simulation domain consists of a rectangle grid with the mesh spacing in all directions chosen to be *10* *nm*. In all the FDTD simulations, we assume the wavelength to be *λ* = *1.55* *μm*. Correspondingly, the permittivities of copper (Cu), silicon (Si) and silica (SiO_2_) are respectively *−67.883* + *10.015i, 12.085* and *2.0851* (ref. 29), which are the materials used in this work. Importantly, the effective mode indices of different waveguide sections are calculated using mode solver simulations (FDTD Solutions mode source), and mesh size of 10 nm is employed for x- and y-directions.

## Additional Information

**How to cite this article**: Liang, Y. *et al*. Integratable quarter-wave plates enable one-way angular momentum conversion. *Sci. Rep.* **6**, 24959; doi: 10.1038/srep24959 (2016).

## Supplementary Material

Supplementary Information

Supplementary Movie S1
