# Supplementary material for: Integratable quarter-wave plates enable one-way angular momentum conversion
Source: Sci Rep. 2016 Apr 22;6:24959. doi: 10.1038/srep24959 (PMC4840338; doi:10.1038/srep24959)
Supplement: Supplementary Information [file srep24959-s1.pdf]

Integratable quarter-wave plates enable one-way angular momentum conversion

Yao Liang[1,2,*], Fengchun Zhang[1,3,*], Jiahua Gu[1], Xu Guang Huang[1] & Songhao Liu[1,3]

[1]Guangdong Provincial Key Laboratory of Nanophotonic Functional Materials and Devices, South China Normal University, Guangzhou 510006, China. [2]Centre for Micro-Photonics, Faculty of Science, Engineering and Technology, Swinburne University of Technology, Hawthorn, Victoria 3122, Australia. [3]Institute of Opto-Electronic Materials and Technology, South China Normal University, Guangzhou 510631, China. Correspondence and requests for materials should be addressed to X.G.H. (email: huangxg@scnu.edu.cn). *These authors contributed equally to this work.

Supplementary Text

1 The quasi-TE and -TM modes in the nanophotonic silicon waveguide.

In nanophotonic waveguides, the longitudinal electric field component (E_z) cannot be ignored, since it accounts for around 34% of total energy. Figure S1a provides the intensity ($I \sim |E|^2$) and phase (Φ) distributions of different polarization components of a quasi-TM mode in the nanophotonic silicon waveguides. Provided the ratio $I_x : I_y : I_z \approx 64\% : 2\% : 34\%$, it is clear the y-polarized component (I_y), which results from the finite size of the Si waveguide in the y-direction, is negigible since it contributes to only about 2% of overall energy.

As for the quasi-TM mode, the light fields along the y-axis is purely transverse, where the dominant electric field component $\boldsymbol{E_x}$ reaches its peaks while other components ($\boldsymbol{E_y}$ and $\boldsymbol{E_z}$) equal zero, meaning that light fields at the y-axis are linearly polarized, as shown in Fig. S1d and S1e. Remarkably, in other points except the ones on the y-axis, the light fields are elliptically polarized, thereby they have both left-handed ($+\sigma$) and right-handed ($-\sigma$) circular polarization components, more precisely, in an unequal manner. Generally, an elliptical polarization can be decomposed into a linear polarization and a circular polarization, and the circularly polarized component is of great

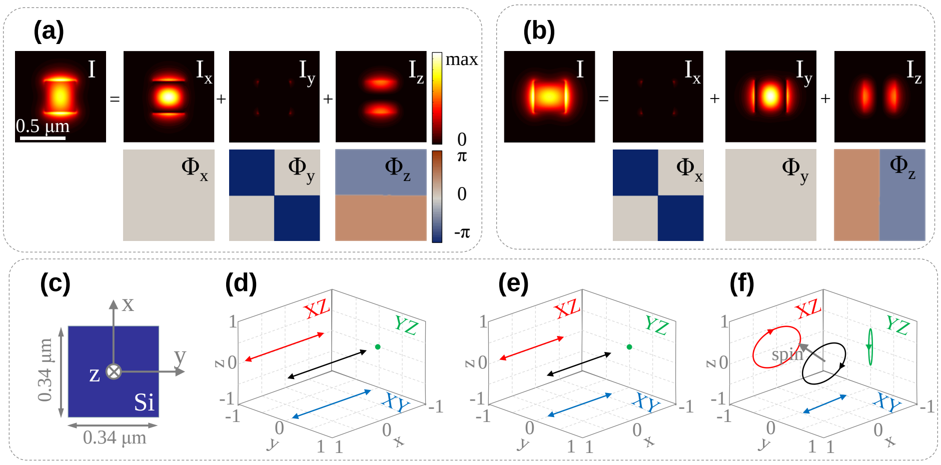

Figure S1. Intensity and phase profiles for (a) the fundamental quasi-TM $|Si,0\rangle$, (b) quasi-TE modes $|Si,\pi/2\rangle$, and their decomposed components in the silicon square waveguide. (c) Geometric details of the Si square waveguide surrounded by silica, which is not shown for clarity, and the coordinate system used. (d), (e), (f) Calculated light fields (E-fields, shown in black) distribution for the quasi-TM mode $|Si,0\rangle$ at points (x, y) = (0, 0), (0, 0.1μm), (0.1μm, 0.1μm), respectively, and their projections in the xy- (blue), xz- (red) and yz-planes (green), with arbitrary units.

interest for the spin of light. Interestingly, the spin AM related to the circularly polarized component is purely transverse, being perpendicular to the propagation direction (positive z-axis). To illustrate this point, we plot the electric field of light at an arbitrary point (x, y) = (0.1 μm, 0.1 μm) in Fig. S1f. It is notable that the electric field projection in the xy-plane is a line while its projections in other planes, such as the xz- and yz-planes, are ellipses, which can be interpreted as a manifestation of the transverse spin. During the preparation of this manuscript, we became aware of a recent work that discussed a new concept named the quantum spin Hall effect (QSHE) of light[1]. In particular, the transverse spin in evanescent waves was demostrated to be the manifestation of the QSHE of light[1].

Similar situations can be found when it comes to the quasi-TE mode, the intensities of which are shown in Fig. S1b. In this case, the light fields are linearly polarized along the x-axis while they are elliptically polarized in other positions. Therefore, the polarization state of photons depends on the position in the transverse plane (xy-plane) in the Si square waveguide. In particular, the light field at the central point ((x, y) = (0, 0)) of the Si square waveguide is purely transversal, regarding to the fundamental 0th order modes (quasi-TE and -TM modes). In the following section, we will further demonstrate that the transverse light field at the central point still holds even in the case of a quasi-circularly polarized mode. Thereby it is reasonable to use the polarization state at the central

point to represent the dominant polarization and phase of a certain fundamental mode.

2 Extraordinary spin angular momentum states of the quasi-circularly polarized mode and the generation of longitudinal vortex mode.

A combination of the quasi-TE and -TM modes (with a relative phase of π/2) results in a quasi-circularly polarized mode with a longitudinal vortex component. Its intensity and phase distributions of different components are shown in Fig. S2a. In particular, the generation of the longitudinal vortex component arises from the superposition of the z-components of the quasi-TE and -TM modes, the schematic of which is shown in Fig. S2b.

Interestingly, the spin of photons at the central point of the Si waveguide is collinear with the propagation direction, as the light field at that point is transversal and circularly polarized (Fig. S2d). In general, however, the spin of photons is neither parallel nor perpendicular to the propagation direction at other positions. To aid interpretation of this finding, in Fig. S2e and S2f, we have respectively plotted the electric field distributions of light at arbitrary points (0, 0.1μm) and (0.1μm, 0.1μm) in the xy-plane, which are elliptically polarized. Their projections in the xy-, yz- and xz-planes imply that unlike the linearly polarized modes (quasi-TE and -TM modes), the spins of a

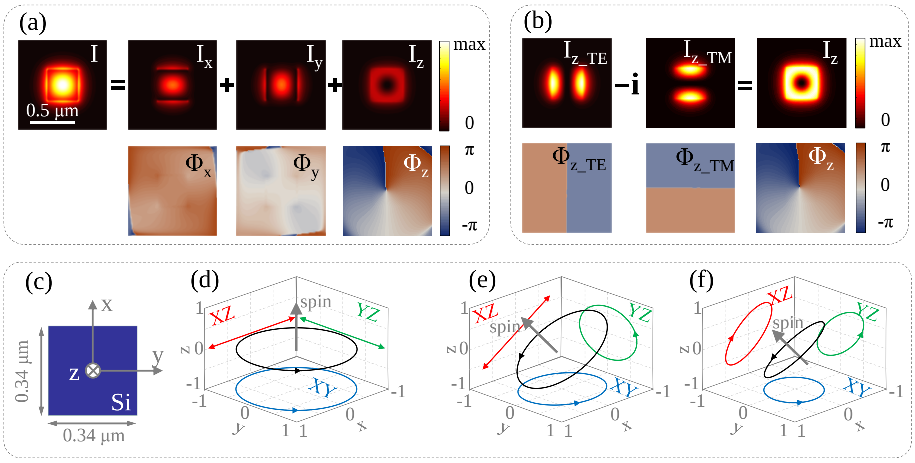

Figure S2. (a) Intensity and phase profiles for a right-handed quasi-circularly polarized mode $|Si,-\sigma\rangle$ and its decomposed components in the Si square waveguide. (b) The schematic of the generation of a longitudinal vortex mode: the superposition of the z-components of the quasi-TE and -TM modes (with a relative phase of π/2), and their corresponding intensity and phase distributions. (c) Geometric details of the Si square waveguide surrounded by silica that is not shown for clarity, and the coordinate system used. (d), (e), (f) Calculated light fields (E-fields, shown in black) distribution for the right-handed quasi-circularly polarized mode $|Si,-\sigma\rangle$ at points (x, y) = (0, 0), (0, 0.1μm), (0.1μm, 0.1μm), respectively, and their projections in the xy- (blue), xz- (red) and yz-planes (green), with arbitrary units.

quasi-circularly polarized mode are usually extraordinary at most positions of the xy-plane, being neither parallel nor perpendicular to the propagation direction. Such extraordinary spin states are expected to hold great potential for a variety of applications. For example, it could be used as the light source that can stimulate Cherenkov surface plasmon waves for metamaterials[2].

3 The handedness consistency of the quasi-circularly polarized modes and their longitudinal twisted components.

There are two kinds of quasi-circularly polarized modes in the Si square waveguide, namely, the left- and right-handed quasi-circularly polarized modes ($|Si,+\sigma\rangle$ and $|Si,-\sigma\rangle$). Due to the strong confinement of light fields in the transversal direction, spin to orbital AM conversion occurs in the Si square waveguide. Consequently, a quasi-circularly polarized mode is to accompany with a longitudinal optical vortex component, the schematic of which are shown in Fig. S3a and S3d.

A left-handed circular polarized mode $|Si,+\sigma\rangle$ results from the superposition of a quasi-TM mode $|Si,0\rangle$ and a quasi–TE mode $|Si,\pi/2\rangle$ that with a phase lag of π/2, that is, $|Si,+\sigma\rangle \sim |Si,0\rangle + i|Si,\pi/2\rangle$ (Fig. S3a). In this case, the light field exhibits a perfect left-handed circular polarization at the central point of the Si waveguide, spinning clockwise (from the point of view of receiver), and the spin points in the counter propagation direction (-z), as shown in Fig. S3c.

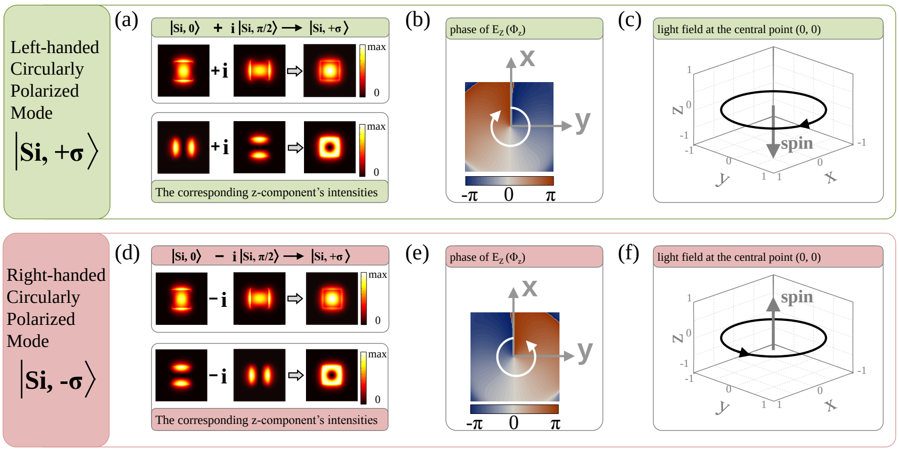

Figure S3. (a) and (d) The intensity profiles of the left- and right-handed quasi-circularly polarized modes ($|Si,+\sigma\rangle$ and $|Si,-\sigma\rangle$) in the Si nanophotonic waveguide, and their decomposed components (above). The corresponding longitudinal components are shown below. (b) and (e) The spatial phase distributions of the longitudinal components (E_z) of the $|Si,+\sigma\rangle$ and $|Si,-\sigma\rangle$ modes, which can be described as $\exp(\pm i\theta)$, where the sign $\pm$ depends on the handedness of twisted direction. The white arrows indicate the twisted direction of the E_z components. (c) and (f) Calculated light field (E-fields, shown in black) distributions at point (x, y) = (0, 0) for the $|Si,+\sigma\rangle$ and $|Si,-\sigma\rangle$ modes (shown in black). The corresponding spins are shown in grey.

Interestingly, the phase distribution of the corresponding longitudinal component (***E***$_z$) exhibits clockwise twisted handedness (from the point of view of receiver), shown in Fig. S3b. It is now obvious that the left-handed quasi-circularly polarized mode and its longitudinal component do have one thing in common: the consistency of their handedness. This conclusion holds true when it comes to a right-handed quasi-circularly polarized mode $|Si,-\sigma\rangle$, as shown in Fig. S3e, S3f.

4 Modes coupling between different nanophotonic waveguides.

When light travels from one kind of waveguide to another, modes coupling occurs. We find that the predominant polarization of a certain mode remains unchanged throughout the coupling process as long as such polarization state is supported in both kinds of waveguides.

To aid interpretation of this finding, we plot the coupling process between the hybrid waveguide and the Si waveguide in Fig. S4a. The modes $|H,\pm\pi/4\rangle$ and $|Si,\pm\pi/4\rangle$ are the modes that supported, respectively, in the hybrid waveguide and the Si square waveguide, where *H* and *Si* indicate the hybrid waveguide and the Si square waveguide respectively, and $\pm\pi/4$ represent the

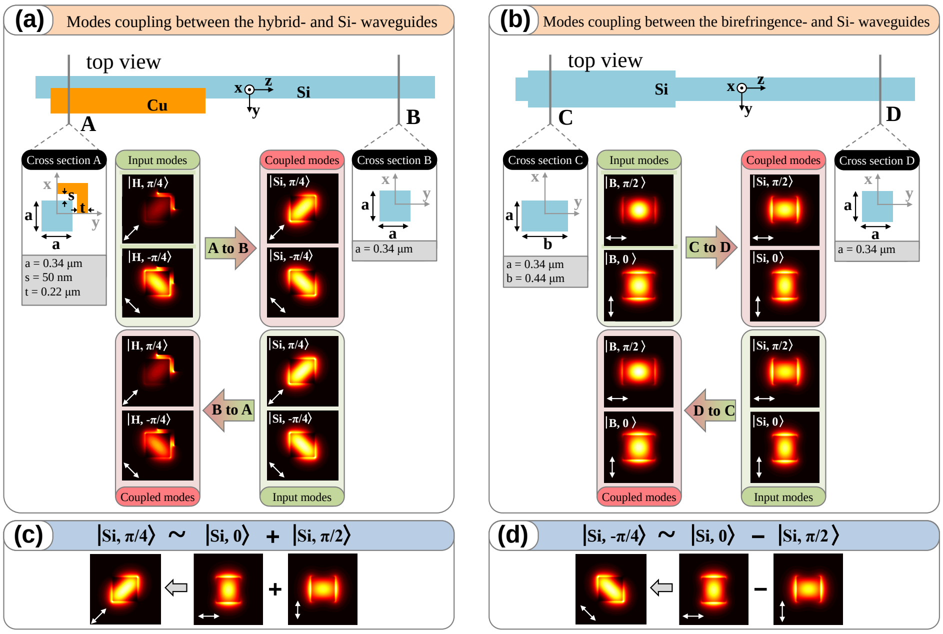

Figure S4. (a) Light fields intensities (I~|E|^2) profiles that reveal modes coupling between the hybrid waveguide and the Si square waveguide. The whole waveguide system is surrounded by SiO_2, which is not shown for clarity. The geometric details of different cross sections and the used coordinates are also included. (b) The modes coupling between the birefringence waveguide and the Si square waveguide. (c) and (d) Light fields intensity profiles of the *±π/4* angles polarized (predominant components) modes and their decomposed components in the Si square waveguide.

predominant polarization components' polarized angles ($\varphi = \pm\pi/4$) down from the positive x-axis. A hybrid waveguide mode $|H,\pm\pi/4\rangle$ can couple into a Si square waveguide mode $|Si,\pm\pi/4\rangle$ when light travels from the hybrid waveguide to the Si square waveguide, and vice versa. As shown in Fig. S4b, similar situations can be found in the coupling processes between the birefringence waveguide and the Si waveguide: the birefringence waveguide modes $|B,0\rangle$ and $|B,\pi/2\rangle$ couple into the Si waveguide mode $|Si,0\rangle$ and $|Si,\pi/2\rangle$, and vice versa, depending on the light's propagation direction, where *B* indicates the birefringence waveguide and *0* and *π/2* are the polarized angles of the predominant polarization components.

It is also notable that a *π/4* angle linearly polarized mode ($|Si,\pi/4\rangle$), whose predominant light field component points along the $\varphi = \pi/4$ direction, results from the superposition of a quasi-TM mode $|Si,0\rangle$ and a quasi-TE mode $|Si,\pi/2\rangle$ in the Si square waveguide, which is, $|Si,\pi/4\rangle \sim |Si,0\rangle + |Si,\pi/2\rangle$, as shown in Fig. S4c. As mentioned in the manuscript, the effective indices of both the quasi-TE and -TM modes are equal ($n_{TE}^{Si} = n_{TM}^{Si} = 2.333$), meaning that these two modes propagate in the same velocity in the Si square waveguide. Consequently, the $|Si,\pi/4\rangle$ mode can remain stable during the propagation. Similarly, a $|Si,-\pi/4\rangle$ mode can be decomposed in a similar way, that is, $|Si,-\pi/4\rangle \sim |Si,0\rangle - |Si,\pi/2\rangle$ (Fig. S4d).

References

1 Bliokh, K. Y., Smirnova, D. & Nori, F. Quantum spin Hall effect of light. *Science* **348**, 1448-1451 (2015).

2 Genevet, P. et al. Controlled steering of Cherenkov surface plasmon wakes with a one-dimensional metamaterial. *Nat. Nanotechnol.* **10**, 804-809 (2015).

Supplementary Movie

Movie S1:

Light fields of a two dimensional (2D) dipole and an in-plane spin dipole on the copper's surface, which are respectively excited by a quasi-linearly mode $\left|Si,\pi/4\right\rangle$ and a right-handed quasi-circularly polarized mode $\left|Si,-\sigma\right\rangle$. The copper thin film is located 400 nm away from the Si nanophotonic waveguide output port and the permittivities of copper (Cu), silicon (Si) and silica (Sio_2) are respectively chosen to be -67.883+10.015i, 12.085 and 2.0851.
